# Detecting Residual Chronic *Salmonella* Typhi Carriers on the Road to Typhoid Elimination in Santiago, Chile, 2017–2019

**DOI:** 10.1093/infdis/jiad585

**Published:** 2023-12-20

**Authors:** Rosanna M Lagos, Michael J Sikorski, Juan Carlos Hormazábal, Alda Fernandez, Sergio Duarte, Marcela F Pasetti, David A Rasko, Ellen Higginson, Joseph Nkeze, Irene N Kasumba, Gordon Dougan, Mailis Maes, Andrew Lees, Sharon M Tennant, Myron M Levine

**Affiliations:** Centro para Vacunas en Desarollo–Chile, Hospital de Niños Roberto del Río, Santiago, Chile; Center for Vaccine Development and Global Health; Center for Vaccine Development and Global Health; Institute for Genome Sciences, Center for Pathogen Research, Department of Microbiology and Immunology, University of Maryland School of Medicine, Baltimore; Sección Bacteriología, Subdepartamento de Enfermedades Infecciosas, Departamento de Laboratorio Biomédico, Instituto de Salud Pública de Chile, Santiago; Sección Bacteriología, Subdepartamento de Enfermedades Infecciosas, Departamento de Laboratorio Biomédico, Instituto de Salud Pública de Chile, Santiago; Sección Bacteriología, Subdepartamento de Enfermedades Infecciosas, Departamento de Laboratorio Biomédico, Instituto de Salud Pública de Chile, Santiago; Center for Vaccine Development and Global Health; Institute for Genome Sciences, Center for Pathogen Research, Department of Microbiology and Immunology, University of Maryland School of Medicine, Baltimore; Center for Vaccine Development and Global Health; Center for Vaccine Development and Global Health; Center for Vaccine Development and Global Health; Cambridge Institute for Therapeutic Immunology and Infectious Disease, University of Cambridge, United Kingdom; Cambridge Institute for Therapeutic Immunology and Infectious Disease, University of Cambridge, United Kingdom; Fina Biosolutions LLC, Rockville, Maryland; Center for Vaccine Development and Global Health; Center for Vaccine Development and Global Health

**Keywords:** typhoid fever, chronic typhoid carriers, transmission of typhoid, typhoid elimination, short-cycle typhoid transmission

## Abstract

**Background:**

In Santiago, Chile, where typhoid had been hyperendemic (1977–1991), we investigated whether residual chronic carriers could be detected among household contacts of non-travel-related typhoid cases occurring during 2017–2019.

**Methods:**

Culture-confirmed cases were classified as autochthonous (domestically acquired) versus travel/immigration related. Household contacts of cases had stool cultures and serum Vi antibody measurements to detect chronic *Salmonella* Typhi carriers. Whole genome sequences of acute cases and their epidemiologically linked chronic carrier isolates were compared.

**Results:**

Five of 16 autochthonous typhoid cases (31.3%) were linked to 4 chronic carriers in case households; 2 cases (onsets 23 months apart) were linked to the same carrier. Carriers were women aged 69–79 years with gallbladder dysfunction and Typhi fecal excretion; 3 had highly elevated serum anti-Vi titers. Genomic analyses revealed close identity (≤11 core genome single-nucleotide polymorphism [SNP] differences) between case and epidemiologically linked carrier isolates; all were genotypes prevalent in 1980s Santiago. A cluster of 4 additional autochthonous cases unlinked to a carrier was identified based on genomic identity (0-1 SNPs). Travel/immigration isolate genotypes were typical for the countries of travel/immigration.

**Conclusions:**

Although autochthonous typhoid cases in Santiago are currently rare, 5 of 16 such cases (31.3%) were linked to elderly chronic carriers identified among household contacts of cases.

## Teaser

Although autochthonous (non-travel-related) typhoid cases in Santiago, Chile, are currently rare compared to the 1977–1991 hyperendemic era, 5 of 16 recent cases (2017–2019) were linked to 4 elderly female chronic carriers by household investigations and whole genome sequencing of isolates.

Low- and middle-income countries where typhoid fever is endemic face a crisis as extensively drug-resistant (XDR) *Salmonella* Typhi (hereafter “Typhi”) are spreading [1, 2]. Only 1 oral antibiotic (azithromycin) remains effective [2]. Accordingly, the global community is mobilizing to control typhoid by prevention. With funding from Gavi, the Vaccine Alliance, many typhoid-endemic countries are implementing programmatic immunization with efficacious single-dose Vi conjugate typhoid vaccines (Vi polysaccharide covalently linked to carrier protein) to render their populations immune [3].

When health authorities in the preantibiotic era controlled endemic typhoid by treating water supplies with sand filtration and/or chlorination to interrupt waterborne long-cycle transmission [4], attention shifted to identify and monitor chronic typhoid carriers (eg, “Typhoid Mary” of early 20th-century New York) [5]. Until chronic carriers died out from the population [6, 7], they constituted a persisting reservoir of Typhi that could propagate short-cycle transmission to proximal contacts who ingested carrier-contaminated food.

During 1977–1991, Santiago, Chile, exhibited enigmatic hyperendemic typhoid in a metropolitan region where approximately 96% of the population, including typhoid cases, had access to treated water supplies and about 80% had toilets connected to a sewer system [8]. Adults in 1980s Santiago also had high prevalences of cholelithiasis and chronic typhoid biliary carriers [9]. Between 1982 and 1991, specific stepwise interventions decreased the annual typhoid incidence by approximately 95% [8, 10].

Strictly defined, chronic typhoid carriers are asymptomatic individuals who persistently excrete Typhi for at least 12 months [11]. Also accepted as chronic carriers are persons from whom Typhi is isolated from bile during cholecystectomy [12, 13]; asymptomatic adult Typhi excreters among household contacts of an acute typhoid fever case in a nonendemic region [14]; and asymptomatic excretion of Typhi by a food handler detected in relation to a foodborne typhoid outbreak investigation, particularly if that individual has an elevated titer of serum Vi antibody [15, 16].

Since Santiago’s chronic carrier prevalence in the hyperendemic era was well-quantified [9], the question arose as to what extent the rare typhoid cases that still occur in Santiago might be linked to residual chronic biliary carriers who were originally infected during the hyperendemic era. To answer this question, we performed epidemiologic investigations of the households of acute autochthonous typhoid fever cases occurring during 2017–2019 to detect elderly chronic carriers responsible for cases via short-cycle transmission.

## METHODS

### Ethics

The household study protocol was approved by the University of Maryland, Baltimore's Institutional Review Board (HP-00083349) and Servicio de Salud Metropolitano Norte's Ethics Committee.

### Definitions

“Cases” are Santiago residents who yielded Typhi in cultures performed in Santiago clinical laboratories, 1 January 2017–31 December 2019, irrespective of medical circumstances that prompted specimen collection. “Autochthonous cases” are Santiago residents who had not traveled abroad within 30 days before onset of clinical illness and were not recent immigrants. “Foreign travel cases” visited typhoid-endemic countries within 30 days of onset of illness; “immigration cases” developed typhoid within 30 days of immigration to Santiago from typhoid-endemic countries. “Household contacts” are persons who shared a domicile and food with a case during ≥3 days over the 2 weeks prior to the date of the index case culture that yielded Typhi, or persons living in the domicile on date of case enrollment.

### Enrollment and Consent

Consented subjects were interviewed to verify and expand information from surveillance forms. These subjects (or parents of pediatric cases) delineated the household demographic composition, provided details for contacting adult members, and authorized seeking informed consent from other household members. Contacts were interviewed about their medical history with emphasis on eliciting evidence of chronic gallbladder disease in adults.

### Contact Stool Cultures

Household contacts of typhoid cases provided stool specimens on at least 3 (to 5) consecutive days to detect Typhi. Fecal swabs in Cary–Blair medium were transported in chilled safety containers to the Instituto de Salud Pública de Chile (ISP) within 24 hours, via courier. Swabs were plated onto MacConkey, *Salmonella-Shigella*, and bismuth-sulfite agars and incubated at 37°C for 48 hours [17, 18]. Suspicious colonies were inoculated into triple-sugar-iron agar slants. Suspect Typhi slant cultures were verified by agglutination with specific typing sera [18]. Typhi colonies were agglutinated by anti-Vi, anti-Group D, and anti-H(d).

### Quantitative Polymerase Chain Reaction for Detecting Typhi

Fecal material suspended in phosphate-buffered saline at room temperature (15°C–25°C) was centrifuged according to Qiagen EZ1 Tissue Kit instructions for preparation of stool specimens. A 200-μL aliquot of liquid phase was incubated with 50 μL of lysis buffer at 56°C. Subsequently, 5 μL of 1 pg/μL phocine herpesvirus DNA control was added with 100 μL elution volumes. Quantitative polymerase chain reaction (qPCR) diagnostics were performed using probesets against 3 targets: *oriC* (common to all *Salmonella* serovars), STY0201 (*staG*, unique to Typhi), and phocine herpesvirus (the control for DNA extraction efficiency and qPCR) (Table 1) [19–21]. The *oriC* and *staG* probesets were previously validated for specificity against DNA from 11 Typhi, 17 other *Salmonella* serovars, and 10 other invasive bacterial species [21]. qPCR was performed in duplicate using 5-μL aliquots with an ABI 7500 Fast system (ThermoFisher) and was used as an endpoint PCR with a quantification cycle of 35 [21].

**Table 1.** Primers and Probes Used for Quantitative Polymerase Chain Reaction to Detect Typhoidal *Salmonella*

Oligo	Sequence (5′ to 3′) and Fluorophore	Target	Reference
oriC-probe	FAM-TGATCTTCAGTGTTTCCCCAACCTGTTTTG-QSY	*Salmonella enterica* (all serovars)	[21]
oriC-F	AGCCAAATCTCCGCTGGAT	*Salmonella enterica* (all serovars)	[21]
oriC-R	CGGAACTGAAAGGCGCTG	*Salmonella enterica* (all serovars)	[21]
ST-probe	VIC-CATTTGTTCTGGAGCAGGCTGACGG-QSY	*Salmonella* Typhi *staG* (STY0201)	[19]
ST-Frt	CGCGAAGTCAGAGTCGACATAG	*Salmonella* Typhi *staG* (STY0201)	[19]
ST-Rrt	AAGACCTCAACGCCGATCAC	*Salmonella* Typhi *staG* (STY0201)	[19]
PhHV-probe	ABY-TTTTTATGTGTCCGCCACCATCTGGATC-QSY	Recombinant pCR TOPO 2.1 gB	[19]
PhHV-Frt	GGGCGAATCACAGATTGAATC	Recombinant pCR TOPO 2.1 gB	[19]
PhHV-Rrt	GCGGTTCCAAACGTACCAA	Recombinant pCR TOPO 2.1 gB	[19]

Abbreviations: PhHV, phocine herpesvirus.

### Whole Genome Sequencing

Typhi DNA from acute case and household contact isolates was sequenced on Illumina HiSeq (Wellcome Sanger) or NextSeq (SeqCenter). Epidemiologically linked isolates were sequenced using Oxford Nanopore Technology (ONT) flow cells. Demultiplexing, quality control, adapter trimming, and base-calling are described in the Supplementary Methods. Genotypes were determined using GenoTyphi v1.9.1 [22]. To generate a local-rooted maximum-likelihood (ML) phylogeny, reads were assembled, and core genome single-nucleotide polymorphisms (SNPs) were identified relative to 2017 Chilean Typhi reference isolate 1521-2017_CI (GenBank accession number CP120397). After removing recombination sites, ML phylogeny was inferred using generalized time-reversible site-substitution model with a Gamma rate distribution and Lewis ascertainment bias correction (ASC_GTRGAMMA) and 100 bootstrap pseudo-replicates. Hybrid complete genome assembly with Illumina and ONT reads was performed. Sequence and assembly accessions are in GenBank BioProject PRJNA935358 and PRJEB20778 (Supplementary Table 1). Pairwise SNP distances between genomes were calculated and compared among epidemiologically linked isolates (Supplementary Table 2). Detailed genomic methods are shown in the Supplementary Methods.

### Antimicrobial Resistance

Phenotypic antimicrobial susceptibility testing (AST) of Typhi isolates was determined by standard disk diffusion method, and genotypic AST was performed on assembled genomes using Pathogenwatch (Supplementary Methods; Supplementary Table 1).

### Vi Antibody

Adult contacts whose stool cultures yielded Typhi provided approximately 5 mL of blood for serum immunoglobulin G (IgG) Vi antibody measurement using an enzyme-linked immunosorbent assay (ELISA) in which biotinylated purified Vi capsular polysaccharide was bound to streptavidin-coated microtiter plates. This assay, “In-house ELISA 6” in Rijpkema et al [23], was established before the International Standard Serum was developed by the National Institute for Biological Standards and Control [23], and US National Institute of Child Health and Human Development IgG Reference reagent Vi-IgG_R1_ was still widely serving as a de facto international standard [24]. Titers are expressed as micrograms of IgG anti-Vi/mL using Vi-IgG_R1_ Reference as the IgG standard [24]. Titers ≥30 μg/mL were considered diagnostic of the chronic typhoid carrier state in persons who have not received Vi-based typhoid vaccines [25]. Vi vaccines were never used as public health tools in Chile. In a parallel 2017–2019 cholecystectomy study carried out in Santiago involving culture and qPCR of bile and pulverized gallstones, 0 of 986 Santiago adults aged 18–34 years and only 2 of 1147 Santiago subjects aged ≥55 years had Vi titers ≥30 μg/mL [25]. No subject in the older or younger age group of the cholecystectomy study grew Typhi in bile, but 1 of the 2 older subjects with a highly elevated Vi titer (≥30 μg/mL) had qPCR evidence of Typhi in pulverized gallstone [25].

## RESULTS

**Figure 1.**
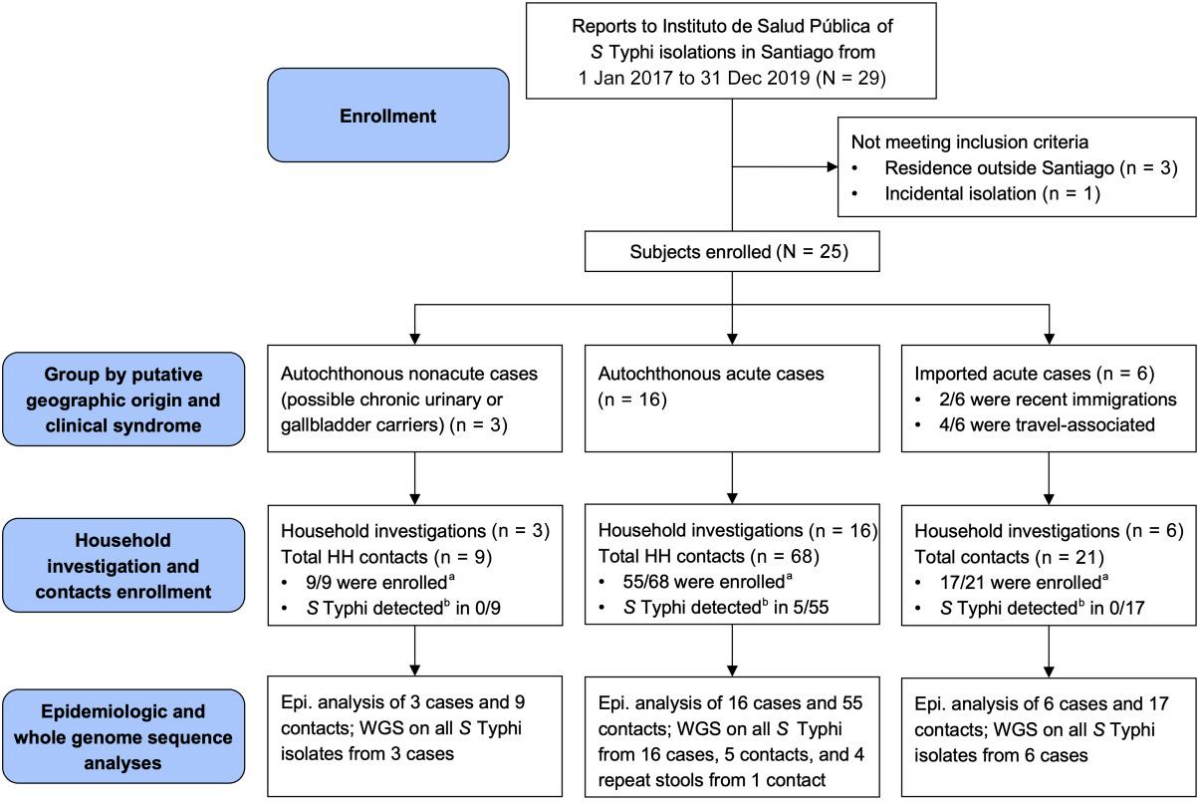
Workflow diagram of study enrollment, follow-up, and analysis of the epidemiologic investigations of typhoid case households to look for chronic carriers and concomitant clinical and subclinical cases. ^a^Household contacts who gave consent and provided stool specimens were enrolled. ^b^Detection in 1 or more stool specimens by both culture and quantitative polymerase chain reaction. Abbreviations: Epi., epidemiological; HH, household; *S* Typhi, *Salmonella enterica* serovar Typhi; WGS, whole genome sequencing.

The case-household study workflow is summarized in Figure 1. Cases were identified at ISP from information provided by clinical laboratories in notification forms accompanying referred Typhi isolates. Surveillance reports from January 2017 to December 2019 revealed 25 Typhi isolations that met study criteria (Figure 1). The case series encompasses 25 Santiago residents who yielded Typhi in a clinical culture, including 19 autochthonous and 6 travel-related or immigration-related cases. The 19 autochthonous cases were reviewed by clinical presentation to glean 16 acute typhoid fever cases (Table 2) including 7 adults (18–49 years) and 9 children (4 aged 10–17 years, and 5 aged <10 years). Fourteen of these 16 acute cases had typical uncomplicated typhoid (multiple days or weeks of fever, malaise, headache [adults, older children], and gastrointestinal complaints). Two children aged <2 years manifested complicated typhoid fever including a 12-month-old infant (index case [ID] 2, Table 2) who experienced several weeks of fever of unknown origin until a stool culture yielded Typhi, and a 9-month-old (ID5, Table 2) who presented with clinical septic arthritis and grew Typhi from aspirated purulent ankle joint fluid. Typhi was cultured from blood of the other 14 cases.

**Table 2.** Results of Epidemiological Investigations of Households of the 16 Autochthonous Acute Clinical Typhoid Fever Cases in Santiago, Chile, Occurring January 2017 Through December 2019 to Detect Chronic Carriers Among Household Contacts

Index Case									Household Contacts						
Case ID	Lab ID	Date of Isolation	Age	Sex	Clinical Specimen	Country of Birth/Recent Travel	Presumed Acquisition of Infection	Date of Household Investigation	Contacts Sampled/Total Contacts	Sex	Age	Stool Specimens (No. Positive/No. Tested)		History or Presence of Gallstones	Serum IgG Anti-Vi Vi Titer of Elderly Household Contact Excreting *S* Typhi
												Positive Culture	Positive qPCR		
1	212-2017	22-01-2017	22 y	M	Blood	Chile/None	Autochthonous	Mar 2019	2/4	F F F F	64 y 55 y 23 y 85 y	0/3 0/3 - -	0/3 0/3 - -	Y; US ’18 N - -	…
2	314-2017	28-01-2017	12 mo	F	Stool	Chile/None	Autochthonous	Jan 2019	5/5	**F** M F F F	**69 y** 40 y 14 y 55 y 54 y	**3/3** 0/3 0/3 0/3 0/3	**3/3** 0/3 0/3 0/3 0/3	**Y; US ’19** N N N N	**682.6 μg/mL**
3	770-2017	18-03-2017	13 y	M	Blood	Chile/None	Autochthonous	Jul 2019	2/2	F F	51 y 74 y	0/3 0/3	0/3 0/3	N N	…
4	1521-2017	02-06-2017	21 y	F	Blood	Chile/None	Autochthonous	Feb 2019	4/4	**F** M M M	**70 y** 2 y 29 y 9 y	**2/2** 0/3 0/3 0/3	**2/2** 0/3 0/3 0/3	**Y; US ’19** N N N	**251.9 μg/mL**
5^a^	1698-2017	29-06-2017	9 mo	F	Joint fluid	Chile/None	Autochthonous	Jan 2019	6/6	**F** F M F **F** M	**77 y** 26 y 27 y 2 y **6 y** 78 y	**2/3** 0/3 0/3 0/3 **1/3** 0/2	**3/3** 0/3 0/3 0/3 **1/3** 0/2	**Y; US ’19** N N N N Y; C ’16	**3.9 μg/mL** ND (child)
7	246-2018	16-01-2018	6 y	F	Blood	Chile/None	Autochthonous	Mar 2019	4/4	F M M M	43 y 44 y 19 y 13 y	0/3 0/3 0/3 0/3	0/3 0/3 0/3 0/3	N N N N	…
12	246-2019	17-01-2019	26 y	M	Blood	Chile/None	Autochthonous	Apr 2019	1/2	F M	67 y 60 y	0/3 -	0/3 -	Y; C ’13 N	…
13	331-2019	23-01-2019	26 y	M	Blood	Chile/None	Autochthonous	Feb 2019	2/2	F F	4 y 25 y	0/3 0/3	0/3 0/3	N	…
15^a^	424-2019	02-02-2019	13 y	M	Blood	Chile/None	Autochthonous	Feb 2019	10/13	F F M M M F F^b^ F^b^ M^b^ F^b^ F^b^ F^c^ M^b^	56 y 1 y 50 y 19 y 30 y 2 y 77 y 26 y 27 y 2 y 6 y 2 y 78 y	0/2 0/3 - - - 0/3 2/3 0/3 0/3 0/3 1/3 0/3 0/2	0/2 0/3 - - - 0/3 3/3 0/3 0/3 0/3 1/3 0/3 0/2	N N - - - N Y; US ’19 N N N N N Y; C ’16	**3.9 μg/mL** ND (child), see case 5
16	1136-2019	28-03-2019	6 y	M	Blood	Chile/None	Autochthonous	Apr 2019	3/3	F M F	30 y 31 y 4 mo	0/3 0/3 0/3	0/3 0/3 0/3	Y; C ’13 N N	…
17	1173-2019	05-04-2019	15 y	M	Blood	Chile/None	Autochthonous	Apr 2019	1/5	F M F F M	44 y 43 y 26 y 2 y 9 y	0/3 - - - -	0/3 - - - -	N - - - -	…
18	2027-2019	19-07-2019	6 y	F	Blood	Chile/None	Autochthonous	Sep 2019	4/4	**F** F M F	**73 y** 29 y 35 y 10 y	**5/5** 0/5 0/1 0/3	**5/5** 0/5 0/1 0/3	**Y; US ’19** N N N	**32.2 μg/mL**
20	2237-2019	16-08-2019	32 y	M	Blood	Chile/None	Autochthonous	Sep 2019	3/3	M F F	65 y 63 y 37 y	0/5 0/5 0/3	0/5 0/5 0/3	Y; C ’02 N N	…
21	2322-2019	27-08-2019	31 y	M	Blood	Colombia/None	Autochthonous	Sep 2019	3/3	F M M	35 y 11 y 5 y	0/5 0/5 0/4	0/5 0/5 0/4	Y; C ’16 N N	…
23	2362-2019	03-09-2019	49 y	F	Blood	Chile/None	Autochthonous	Sep 2019	3/5	M F F F M	47 y 18 y 74 y 47 y 45 y	0/5 0/5 0/5 - -	0/5 0/5 0/5 - -	N N N - -	…
25	3273-2019	18-12-2019	21 mo	M	Blood	Haiti/None	Autochthonous	Jan 2020	2/3	F F M	22 y 25 y 22 y	0/5 0/5 -	0/5 0/5 -	N N N	…

Case ID = case number assigned in chronological order; Lab ID = laboratory sample identifier; Date of isolation = day-month-year; Age of the case = years or months if <2 years of age; Clinical specimen = site of culture collection; Country of birth = self-reported; Recent travel = foreign travel within 30 days prior to clinical presentation; Presumed acquisition of infection = determination made by study team based on all available epidemiologic information collected through household investigations; Contacts sampled/total contacts = the number of household contacts who gave consent and stool specimens over the total number of household contacts reported (including absent or nonconsenting contacts); Sex = biological sex of household contact reported by self or family member; Age = age of contact reported by self or family member; Stool specimens (No. positive/No. tested) = the number of stool specimens positive for *Salmonella* Typhi by qPCR or culture over the total number of stool specimens collected; History/presence of gallstones = history or presence of gallstones (cholelithiasis), Yes (Y) or No (N), if yes then confirmed by ultrasound or cholecystectomy and date abbreviated into a 2-digit format (’YY); Serum anti-Vi IgG titer = serum anti-Vi IgG titer measured in adult carriers by Center for Vaccine Development and Global Health enzyme-linked immunosorbent assay using US reference reagent Vi-IgG R1, 201 from the US National Institute of Child Health and Human Development. Serum IgG Vi titer was not measured in the 6-year-old stool culture–positive household contact of case 5. Bold text indicates a chronic typhoid carrier.

Abbreviations: C, cholecystectomy; F, female; IgG, immunoglobulin G; M, male; ND, not done; qPCR, quantitative polymerase chain reaction; US, ultrasound; -, not available for testing.

^a^Case 5 (1698-2017) and case 15 (424-2019) are cousins who reside in 2 different households in distinct parts of Santiago. Case 15 became ill with typhoid after spending approximately 3 weeks living in the household of his great-grandmother where case 5 resides permanently.

^b^Denotes the temporary household contacts of case 15 during his stay at the great-grandmother's house where case 5 resided permanently. These “temporary” (for several weeks) household contacts had already provided stool samples for the investigation of case 5 a few weeks earlier, so they were not asked to provide additional samples for the investigation of case 15.

^c^This temporary household contact of case 15 was herself a typhoid case (case 5) 20 months earlier.

The 16 autochthonous typhoid cases included 2 children who were consanguineous cousins (Table 2, ID5 and ID15) residing in different Santiago districts who had onset of typhoid 23 months apart. Case ID5 lived in a household with a 77-year-old great-grandmother with chronic gallbladder disease who had multiple stools positive for Typhi (culture and qPCR); a 6-year-old asymptomatic sister had 1 positive stool culture. The cousin (ID15) spent several weeks at this great-grandmother's home before developing illness.

Besides 16 autochthonous acute typhoid fever cases, there were 3 Typhi isolations from adult Santiago residents (ID10, ID11, ID14) who lacked recent travel and did not manifest typical acute typhoid clinically (Table 3). Because of their unusual clinical presentations, medical histories, and the clinical specimens from which their Typhi were isolated (which are distinct from typical clinical acute typhoid fever patients), cases 10, 11, and 14 are deemed to be chronic typhoid carriers; 10 as a urinary carrier and 11 and 14 as biliary carriers (Table 3). ID10 was a 47-year-old man with intermittent episodes of abdominal pain and vomiting whose urine grew Typhi (possible urinary carrier). ID11 was a 65-year-old man with a history of symptomatic cholelithiasis who was awaiting elective cholecystectomy when he developed an “acute abdomen” from gallbladder rupture; bilious peritoneal fluid from surgery grew Typhi. ID14, a 26-year-old female Haitian immigrant with chronic renal disease living in Santiago since 2017, grew Typhi from a coproculture while hospitalized in 2019 because of severe abdominal pain and vomiting (diagnosed as acute pancreatitis). No tested household contacts of these 3 cases yielded Typhi in stools.

**Table 3.** Autochthonous *Salmonella* Typhi Infections of Individuals Who Did Not Present Clinically as Acute Typhoid Illness but Rather Appeared to be Chronic Biliary or Chronic Urinary Typhoid Carriers and Results of Epidemiological Investigations to Detect Household Contacts Excreting *S* Typhi

Case ID (Lab ID), Date of Isolation	Index Case					Household Contacts						
	Age and Sex of the Case (Clinical Specimen)	Country of Birth	History of Recent Travel or Immigration	Presumed Place of Acquisition of Infection	Serum IgG Anti-Vi Titer, μg/mL	Follow-up Period	Contacts Sampled/Total Contacts	Sex	Age	Stool Specimens (No. Positive/No. Tested)		History/Presence of Gallstones
										Positive Culture	Positive qPCR	
Case 10 (1329-2018), 25-04-2018	47 y, M (urine)	Chile	No	Chile, autochthonous	281.1	Feb 2019	2/2	F M	68 y 67 y	0/3 0/3	0/3 0/3	Y; C ’70 N
Case 11 (1738-2018), 17-06-2018	65 y, M (bilious peritoneal fluid from ruptured gallbladder in an individual with chronic gallbladder disease who was awaiting cholecystectomy when he presented with an acute abdomen requiring surgical intervention)	Chile	No	Chile, autochthonous	333.6	Feb 2019	6/6	F F F F M M	62 y 34 y 4 y 31 y 9 y 2 y	0/3 0/3 0/3 0/3 0/3 0/3	0/3 0/3 0/3 0/3 0/3 0/3	N N N N N N
Case 14 (426-2019), 01-02-2019	26 y, F (stool)	Haiti	No	Chile, autochthonous	13.9	Feb 2019	1/1	M	39 y	0/3	0/3	N

Case ID = case number assigned in chronological order; Lab ID = laboratory sample identifier; Date of isolation = day-month-year; Age of the case = years or months if <2 years of age; Country of birth = self-reported; Recent travel = foreign travel outside Chile within 30 days prior to clinical presentation; Presumed place of acquisition of infection = determination made by study team based on all available epidemiologic information collected through household investigations; Serum anti-Vi IgG titer = serum anti-Vi IgG titer measured in adult carriers, by enzyme-linked immunosorbent assay, using the US National Institutes of Health Reference Standard reagent Vi-IgG_R1_; Follow-up period = the month of initial household investigation, lasting 2–4 weeks; Contacts sampled/total contacts = the number of household contacts who gave consent and stool specimens over the total number of household contacts reported (including absent or nonconsenting contacts); Sex = biological sex of household contact reported by self or family member; Age = age of contact reported by self or family member; Stool specimens (No. positive/No. tested) = the number of stool specimens positive for *Salmonella* Typhi by qPCR or culture over the total number of stool specimens collected; History/presence of gallstones = history or presence of gallstones (cholelithiasis), Yes (Y) or No (N), if yes then confirmed by ultrasound or cholecystectomy and date abbreviated into a 2-digit format (’YY).

Abbreviations: C, cholecystectomy; F, female; IgG, immunoglobulin G; M, male; qPCR, quantitative polymerase chain reaction.

The 6 travel/immigration-related cases who developed classical typhoid fever arrived in Santiago from a typhoid-endemic country <10 days prior to their confirmatory culture date (Table 4). No stool cultures of household contacts of these cases yielded Typhi.

**Table 4.** Follow-up of 6 Nonautochthonous (Travel-Related or Recent Immigration-Related) Cases of Acute Typhoid Fever and Results of Epidemiological Investigations to Detect Chronic Carriers Among the Household Contacts of These Cases

Index Case					Household Contacts				
Case ID (Lab ID), Date of Isolation	Age and Sex (Specimen)	Country of Birth	Source Country of Immigration-Related Case	Countries Visited by Travel-Related Cases	Follow-up Period	Contacts Sampled/Total Contacts, No.	Sex	Age	Stool Specimens (No. Positive/No. Tested)
Case 6 (28-2018), 28-12-2017	6 y, M (blood)	Haiti	Haiti	…	Jan 2019	5/9	F M M F M F M M F	43 y 48 y 15 y 10 y 10 y 43 y 6 y 34 y 32 y	0/3 - - 0/3 0/3 0/3 0/3 - -
Case 8 (387-2018), 29-01-2018	7 y, F (blood)	Peru	…	Peru	Jan 2019	4/4	F M F M	28 y 41 y 67 y 70 y	0/3 0/3 0/3 0/3
Case 9 (1208-2018), 16-04-2018	8 y, F (blood)	Haiti	Haiti	…	Jan 2019	4/4	F M M M	36 y 34 y 10 y 2 mo	0/3 0/3 0/3 0/3
Case 19 (2224-2019), 15-08-2019	26 y, M (blood)	Chile	…	Mexico, Panama	Oct 2019	1/1	F	33 y	0/4
Case 22 (2319-2019), 29-08-2019	25 y, F (stool)	Peru	…	Peru	Sep 2019	2/2	F M	27 y 54 y	0/5 0/5
Case 24 (2854-2019), 13-11-2019	26 y, F (blood)	Mexico	…	Mexico	Dec 2019	1/1	M	34 y	0/5

Case ID = case number assigned in chronological order; Lab ID = laboratory sample identifier; Date of isolation = day-month-year; Age of the case = years or months, if <2 years of age; Country of birth = self-reported; Source country of immigration-related case was determined by the study team based on all available epidemiologic information collected through household investigations; Follow-up period = the month of initial household investigation, lasting 2–4 weeks; Contacts sampled/total contacts = the number of household contacts who gave consent and stool specimens over the total number of household contacts reported (including absent or nonconsenting contacts); Sex = biological sex of household contact reported by self or family member; Age = age of contact reported by self or family member; Stool specimens (No. positive/No. tested) = the number of stool specimens positive for *Salmonella* Typhi by quantitative polymerase chain reaction or culture over the total number of stool specimens collected.

Abbreviations: F, female; M, male; -, not available for testing.

### Chronic Carriers Detected Within Autochthonous Acute Typhoid Case Households

Investigations of the 16 autochthonous acute typhoid case households identified 68 domestic contacts, of whom 55 provided stool for culture and PCR (Figure 1; Table 2). Five of 55 contacts (9.1%) were excreting Typhi, including 4 elderly women (69–79 years of age); additionally, the 6-year-old sister of ID5 had a positive coproculture on a single day. The 4 elderly afebrile women silently excreting Typhi were permanent or temporary household contacts of 5 of the 16 autochthonous acute typhoid cases (31.3%) including 2 cousins who developed disease 23 months apart, 1 in 2017 and 1 in 2019, following contact with their mutual great-grandmother. One elderly women had cholelithiasis diagnosed prior to enrollment. In the other 3 cholelithiasis was confirmed by abdominal ultrasound examination during this epidemiologic study. Three of these 4 elderly asymptomatic Typhi excreters had IgG Vi antibody titers ≥30 μg/mL (Table 2), indicating chronic Typhi carriage; 2 women's titers were exceptionally elevated (251.9 and 682.6 μg/mL).

### Genotypes, Phylogeny, and SNP Distances

**Figure 2.**
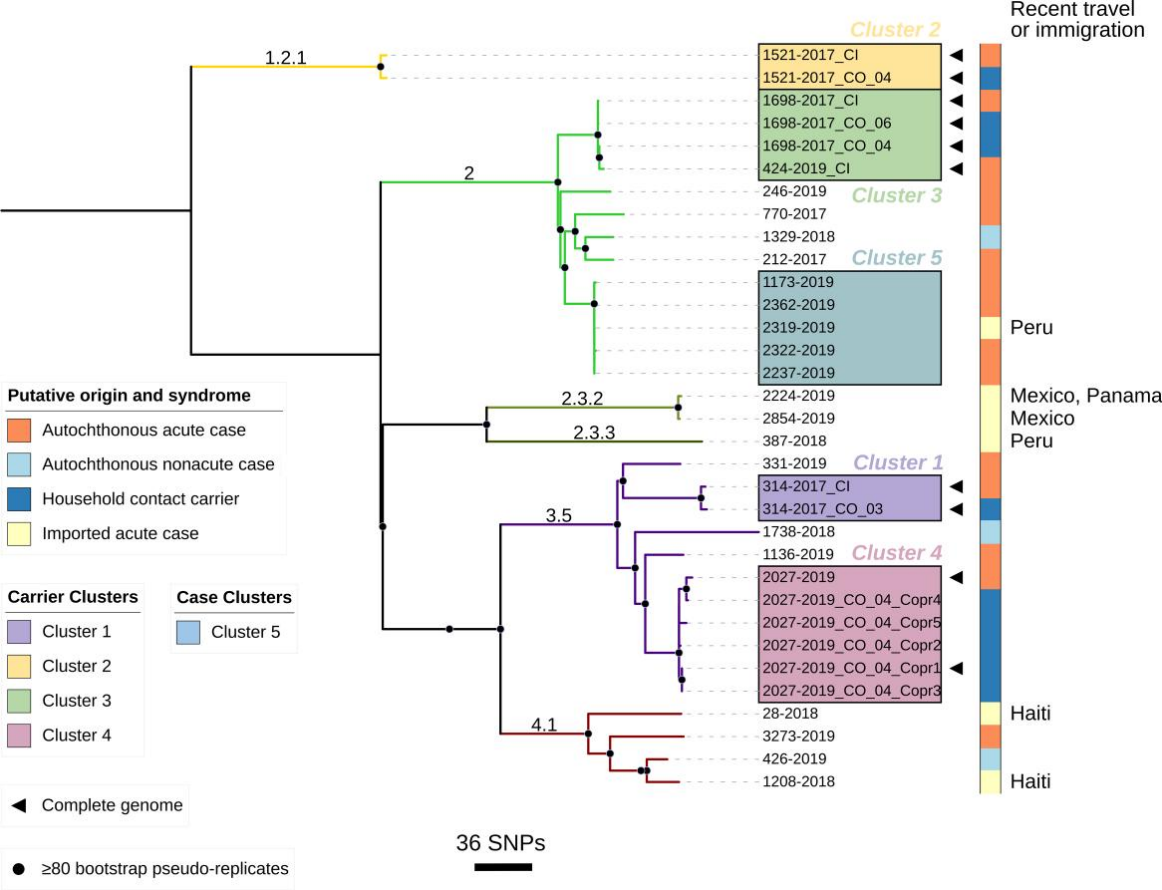
Maximum likelihood tree depicting core genome phylogenetic relationships of *Salmonella* Typhi isolates in this study. Branches are both labeled and colored by GenoTyphi assigned genotypes. Putative geographic origin (autochthonous or imported, and travel/immigration-associated country if imported) and clinical syndrome (acute or nonacute case) are indicated by the color strip. Carrier clusters 1–4, each associated with a confirmed chronic carrier detected through household investigations, are distinguished by both colored boxes and adjacent labels. Completed genomes (assembled from Illumina and Oxford Nanopore reads) are identified by a black triangle. Bootstrap supported nodes are identified by black-shaded circles. The scale bar shows a measure of the number of single-nucleotide polymorphisms (SNPs) by branch length. Tree visualized using iTol v6.7.5 (https://itol.embl.de/).

Maximum likelihood core genome phylogenetic relationships of Typhi isolates from the overall 25 patients are depicted in Figure 2. The 16 autochthonous acute typhoid case isolates include genotypes 2 (9/16 [56.3%]), 3.5 (4/16 [25.0%]), 1.2.1 (1/16 [6.3%]), and 4.5 (1/16 [6.3%]); 1 case isolate (ID7; 246-2018) failed quality control and was excluded from genomic analysis. The 3 autochthonous nonacute atypical case isolates were genotypes 2, 3.5, and 4.1 (Figure 2). The 6 travel/immigration-related acute case isolates include genotypes 2 and 2.3.3 (Peru), 2.3.2 (n = 2, Mexico), and 4.1 (n = 2, Haiti) (Figure 2).

Genotypes of stool isolates (n = 5) from the asymptomatic household/familial contacts were identical to the genotype of their associated index case, yielding 4 household/familial clusters labeled 1–4 (Table 5; Figure 2). The core genome SNP distance within each cluster ranged from 0 to 11 SNPs, with a date difference of isolation ranging from 81 to 731 days. In contrast, each individual cluster was genomically distinct with >50 SNP differences to the nearest external isolate. Five Typhi stool isolates collected over 8 days from the 73-year-old grandmother associated with ID18 were all genotype 3.5 and within 0–8 SNPs difference.

**Table 5.** Analysis of the Complete Genomes of *Salmonella* Typhi Isolates From the Acute Index Cases and Their Household Chronic Carrier in 4 Household/Familial Clusters

Cluster	Clinical Host	Isolate ID^a^	Date of Isolation	Days Between Index Case Isolate and Carrier Isolate^b^	Sex	Age	GenoTyphi Genotype	SNP Distance Between the Index Case Isolate and Carrier Isolate^c^	SNP Distance Between First and Subsequent Coproculture Isolates From Same Carrier	SNP Distance to the Nearest Isolate External to This Cluster
Cluster 1	Index case 2	314-2017_CI	28-01-2017	-	F	13 mo	3.5	…	…	95
	**Chronic carrier**	**314-2017_CO_03**	**29-01-2019**	**731 d**	**F**	**71 y**	**3.5**	**7**	…	**96**
Cluster 2	Index case 4	1521-2017_CI	02-06-2017	-	F	21 y	1.2.1	…	…	293
	**Chronic carrier**	**1521-2017_CO_04** ^d^	**12-02-2019**	**620 d**	**F**	**71 y**	**1.2.1**	**8**	…	**293**
Cluster 3	Index case 5	1698-2017_CI	29-06-2017	-	F	9 mo	2	…	…	52
	Temporary carrier	1698-2017_CO_04	10-01-2019	560 d	F	6 y	2	1	…	53
	**Chronic carrier**	**1698-2017_CO_06** ^d^	**10-01-2019**	**560 d**	**F**	**79 y**	**2**	**0**	…	**52**
	Index case 15	424-2019_CI	02-02-2019	583 d	M	13 y	2	4	…	56
Cluster 4	Index case 18	2027-2019_CI	19-07-2019	-	F	6 y	3.5	…	…	58
	**Chronic carrier**	**2027-2019_CO_04** ^d,e^	**29-09-2019**	**81 d**	**F**	**73 y**	**3.5**	**11**	…	**51**
	Repeat coproculture	_Copro2^f^	30-09-2019	82 d			3.5	10^g^	3	50
	Repeat coproculture	_Copro3^f^	01-10-2019	83 d			3.5	11^g^	0	51
	Repeat coproculture	_Copro4^f^	02-10-2019	84 d			3.5	5^g^	8	55
	Repeat coproculture	_Copro5^f^	07-10-2019	89 d			3.5	14^g^	7	54

Associations are shown in relation to calendar time and SNP distances.

Abbreviations: F, female; M, male; SNP, single-nucleotide polymorphism.

^a^CI = Index case (note that cluster 3 includes 2 index cases (case 5 and case 15) widely separated in dates of onset); CO = Household contact; Copro# = Day of collection of 5 daily consecutive coprocultures from 1 chronic carrier.

^b^The number of days between the isolation of *Salmonella* Typhi from the index acute case and the isolation of *S* Typhi from the stool of the household contact collected during a case investigation.

^c^The number of core genome SNPs difference between the stool culture isolate from a household contact and the index acute case isolate.

^d^The chronic carriers, all women 71–79 years of age, are highlighted in bold.

^e^This chronic carrier within cluster 4 had coprocultures collected on 5 consecutive days to assess SNP differences on multiple days in the same chronic carrier.

^f^Coprocultures 2–5 are draft genomes.

^g^The first number is the SNP distance for each sequential coproculture with respect to the index case, and the following number in parentheses is the SNP distance from coproculture 1 to measure within-host variation over sequential coprocultures.

A fifth cluster (cluster 5, Figure 2) of typhoid cases was detected based on genome sequencing. Four autochthonous (1173-2019, 2362-2019, 2322-2019, and 2237-2019) cases and 1 travel-associated case (2319-2019 [Peru]) occurred from April through September 2019. All 5 isolates were of genotype 2 and virtually identical (0–1 SNP differences). Household investigations of 12 of the 18 household contacts of these cases revealed no asymptomatic carriers.

### Antimicrobial Susceptibility

AST patterns are found in Supplementary Table 1. All isolates except 1 (331-2019) showed phenotypic pan-susceptibility to clinically useful antibiotics including ciprofloxacin. No isolates contained antimicrobial resistance–associated genes. Two isolates (2224-2019 and 2854-2019 associated with recent travel to Mexico) contained a single Q465L point mutation in DNA gyrase B (*gyr*B), although phenotypically these isolates were pan-susceptible including to ciprofloxacin.

### Carrier Follow-up

The 4 chronic carriers were notified when their coproculture results became known and were counseled to avoid new infections within the household by practicing hygienic precautions. Carriers were referred to their healthcare providers. Abdominal ultrasound examinations were arranged for 3 carriers (ID-1521-2017-CO4, ID-1698-2017-CO6, and ID-2027-2019-CO4) who had not previously had ultrasound studies; gallstones were detected in all 3. The fourth carrier, ID-314-2017-CO3, had been diagnosed with cholelithiasis before entering the study and was awaiting cholecystectomy. Carrier ID-1521-2017-CO4 underwent cholecystectomy in February 2022. Carrier ID-1698-2017-CO6, who was not referred to surgery because of senior age, comorbidities, and absence of bothersome gallbladder symptoms, received antibiotics. Her 3 follow-up monitoring stool cultures have remained negative.

## DISCUSSION

From 1982 to 1991, 2 interventions drastically reduced the high annual incidence of typhoid fever in Santiago, Chile. These included large-scale field evaluations of Ty21a live oral typhoid vaccine among approximately 500 000 schoolchildren [8, 26–29], followed by an enforced prohibition against using untreated sewage water to irrigate vegetable crops [30] during Santiago's 1991 cholera outbreak [31, 32]. Cholera transmission was interrupted, and annual warm season typhoid spikes also ceased [8, 10, 31, 32], resulting in control of endemic typhoid and its near elimination in the next few years. Following disappearance of typhoid as a common health problem, attention in Chile turned to the high frequency of gallbladder cancer as an apparent legacy of chronic typhoid gallbladder carriage [33].

Since 2000, typhoid cases in Santiago have been distinctly uncommon and often linked to travel to typhoid-endemic countries, a pattern observed in other high-income countries [34]. During 2017–2019, we sought epidemiologic evidence of autochthonous transmission among the few cases of acute typhoid fever still occurring in Santiago by searching for elderly chronic carriers among household contacts of the sparse autochthonous acute typhoid cases. The expectation is that as these carriers die off in ensuing years, the long-term reservoir of Typhi will correspondingly disappear. This epidemiologic investigation identified 25 culture-confirmed Typhi infections encompassing 6 travel/immigration-related cases and 19 autochthonous cases; 16 autochthonous case isolates were from patients with acute typhoid fever. Although the study size is relatively small, among 55 household contacts of these 16 autochthonous acute typhoid cases, 4 putative chronic biliary carriers were detected who were responsible for 5 of 16 autochthonous cases (31.3%). Each chronic carrier exhibited the classic phenotype of an older (69–79 years) woman with gallbladder disease [9]. These older women excreting viable Typhi had a high likelihood of past exposure to Typhi when they were in their 20s, 30s, and 40s, since they lived in Santiago during the decades of hyperendemic typhoid transmission when confirmed carriers in this age range were prevalent [9, 12]. Each woman had multiple stool specimens positive for Typhi by both culture and qPCR. Vi serology corroborated the chronic carrier status of 3 women, as each had a high anti-Vi IgG titer ≥30 μg/mL [25]. Vi antibody measured by various assays has historically been used to detect carriers responsible for outbreaks [15, 16], or to screen for chronic carriers in populations [35–37]. To our knowledge this is the first time that Vi serology has been used systematically to help confirm most (3 of 4) chronic carriers incriminated in conjunction with rare sporadic typhoid cases in a nonendemic area where Vi-based vaccines are not routinely used. If older adults receive Vi vaccine, anti-Vi titers may be uninterpretable. For example, the Samoa Typhoid Fever Control Program does not administer Vi conjugate to adults >45 years of age [38]. Measurement of antibodies to YncE may be helpful in the search for chronic carriers among adults who have received Vi-based vaccines [39].

Each putative silent chronic carrier and the acute typhoid cases with which they were linked constitute epidemiologic clusters. Genomic analyses of these case-carrier clusters provided evidence supporting epidemiologic data, indicating that chronic carriers transmitted Typhi to susceptible household contacts. Although isolates from chronic carriers were obtained 81–731 days after collection of the clinical specimen that yielded the index case isolate, core genome analyses revealed very close identity between epidemiologically linked case and carrier isolates. Indeed, within each epidemiologic dyad, genotypes of carrier and index case isolates were identical and SNP differences were minimal (1, 7, 8, and 11 SNP differences, respectively). These minor SNP differences indicate short-cycle transmission events [40, 41]. In 1 chronic carrier (contact of ID18), stool isolates collected over 9 days showed 3–8 SNP differences, supporting modest within-host variability. In contrast, the individual case-carrier clusters were distinct from their nearest external isolates by >50 SNPs, indicating a very low probability of relatedness to other autochthonous transmission events.

We do not know the source of typhoid infection for the remaining 11 of 16 autochthonous cases for which a carrier in the household could not be found. However, near identity by whole genome sequencing of 4 isolates of typical Chilean genotype 2 (1173-2029, 2322-2019, 2322-2019, and 2237-2019; Figure 2) suggest they have an epidemiologic linkage such as via food contaminated by a carrier working in a restaurant or food kiosk frequented in common by the cases. The remaining 7 autochthonous acute cases were also infected with typical Chilean genotypes.

Three additional autochthonous Typhi isolations (ID10, ID11, ID14) had clinical histories compatible with chronic forms of Typhi carriage rather than acute infection. ID11, who suffered from chronic gallbladder disease, experienced gallbladder perforation and grew Typhi from peritoneal fluid collected during emergency surgery. Whereas chronic Typhi carriage is most common in the gallbladder, chronic urinary carriers also exist [42–44]. ID10, the 47-year-old man who grew Typhi in urine cultures following 6 months of episodic abdominal pain and vomiting, is compatible with a diagnosis of chronic urinary carriage [44]. Subjects ID10 and ID11 also had extremely high Vi antibody titers (333.6 and 281.1, respectively) consistent with chronic Typhi carriage. The third case (ID14) was an immigrant from Haiti who had Typhi isolated from a stool culture when she sought healthcare because of acute abdominal pain and vomiting. We surmise that ID14 was already a chronic carrier when she immigrated from Haiti and that her carriage was detected serendipitously during a hospitalization for abdominal pain and vomiting that yielded a diagnosis of acute pancreatitis. Her isolate's genotype, 4.1, compatible with Haitian origin, supports this conclusion.

Predominant genotypes among Typhi isolates from acute autochthonous cases during 2017–2019, genotypes 2 and 3.5, comprising 81.3% of 16 cases, match the 2 dominant genotypes detected among Typhi case isolates during the typhoid hyperendemic 1980s [45]. These observations support the contention that historic local Chilean Typhi genotypes have been carried through these decades within the gallbladders (or urinary tracts) of chronic typhoid carriers.

Control programs are under way in many typhoid-endemic countries to diminish typhoid transmission using Vi conjugate vaccine as the key intervention. Additionally, where feasible, we recommend that specialized typhoid epidemiological investigation teams be established to investigate households (and other relevant venues) of non-travel-associated typhoid cases [38, 41, 46]. Chronic carriers, the long-term reservoir of Typhi, will eventually die off from these populations as transmission diminishes [6, 7]. In the interim, carriers with suboptimal hygienic practices who handle food can cause cases among their household contacts, such as the Santiago great-grandmother who over 2 years infected 2 great-grandchildren. Carriers can spread typhoid more extensively if they are commercial food handlers. If chronic carriers are excreting antibiotic-susceptible Typhi, they can be offered antimicrobial therapy [17]. If they are shedding XDR organisms, cholecystectomy is a potential, albeit invasive and expensive, treatment option [7, 47–49]. At the least, wherever possible, chronic carriers excreting XDR Typhi should be monitored by health authorities with annual coprocultures to determine if they are still shedding, and the carriers should be given health education to reinforce the importance of proper handwashing and food handling practices [11]. These interventions can help minimize the propensity for chronic carriers to transmit typhoid.

## Supplementary Data

Supplementary materials are available at *The Journal of Infectious Diseases* online (http://jid.oxfordjournals.org/). Supplementary materials consist of data provided by the author that are published to benefit the reader. The posted materials are not copyedited. The contents of all supplementary data are the sole responsibility of the authors. Questions or messages regarding errors should be addressed to the author.

## Supplementary Material

jiad585_Supplementary_Data

## Notes

***Acknowledgments.*** The authors wish to thank the study participants. The authors thank Professor Nicholas R. Thomsen of the Wellcome Sanger Institute for access to sequencing.

***Author contributions.*** M. M. L., R. M. L., J. C. H., S. M. T., M. J. S., and M. F. P. conceptualized this study. M. J. S., A. F., and J. C. H. were responsible for building and maintaining the study database as well as data curation. M. M. L., R. M. L., M. J. S., D. A. R., J. C. H., M. F. P., and S. M. T. performed the formal analyses. M. M. L. acquired the financial support for the project leading to this publication and managed and coordinated the study. R. M. L., M. M. L., J. C. H., S. M. T., M. J. S., D. A. R., E. H., A. L., J. N., I. N. K., M. M., G. D., and M. F. P. carried out the investigations and developed and validated the methodologies. This project was administered by M. M. L., R. M. L., J. C. H., and M. F. P. Resources, including volunteer subject specimens required for the research, were made available by R. M. L., A. L., D. A. R., G. D., and M. F. P. The original draft of this manuscript was authored by R. M. L., M. M. L., M. J. S., D. A. R., J. C. H., S. M. T., and E. H. No authors were precluded from accessing data from the study and they accept responsibility for publication.

***Data sharing.*** De-identified individual participant data collected during the study as well as study protocols, informed consent forms, and database dictionaries will be provided upon reasonable request by researchers who provide a methodologically sound proposal to achieve aims in the approval proposal. Requests should be directed to the corresponding author (M. M. L.) or secondary corresponding author (M. J. S.); researchers will need to sign a data access agreement.

***Disclaimer.*** The Bill & Melinda Gates Foundation had no role in data collection, analysis, or interpretation; trial design; or patient recruitment. No funds were provided for this research by any pharmaceutical company.

***Financial support.*** Funding for this work was supported by the Bill & Melinda Gates Foundation (grant number OPP1161058; M. M. L., Principal Investigator). Under the grant conditions of the Bill & Melinda Gates Foundation, a Creative Commons Attribution 4.0 Generic License has already been assigned to the Author Accepted Manuscript version that might arise from this submission. M. M. L. is supported in part by the Simon and Bessie Grollman Distinguished Professorship at the University of Maryland School of Medicine; the National Institute of Allergy and Infectious Diseases/National Institutes of Health (NIAID/NIH) (grant number U19-AI142725); and a Wellcome Trust Strategic Translation Award. M. J. S. received research support from the NIAID/NIH (grant number F30AI156973) and the National Institute of Diabetes and Digestive and Kidney Diseases (grant number T32DK067872). Funding to pay the Open Access publication charges for this article was provided by the Bill & Melinda Gates Foundation.
