# Supplementary material for: Detecting Residual Chronic Salmonella Typhi Carriers on the Road to Typhoid Elimination in Santiago, Chile, 2017–2019
Source: J Infect Dis. 2023 Dec 20;230(2):e254–67. doi: 10.1093/infdis/jiad585 (PMC11326835; doi:10.1093/infdis/jiad585)
Supplement: jiad585_Supplementary_Data [file jiad585_supplementary_data.zip › Lagos_etal_TF_Carriers_Suppl_Methods.docx]

SUPPLEMENTAL METHODS

**Whole genome sequencing and assembly.**

Illumina reads were generated from pure bacterial cultures at two sequencing centers. At the Wellcome Sanger Institute (Hinxton, UK), genomic DNA was extracted from *S.* Typhi isolates using the Norgen DNA extraction kit following manufacturer’s instructions and sequenced by Illumina HiSeq 6000 generating 125-bp paired reads. At the SeqCenter (Pittsburgh, PA) samples were pre-treated with lysozyme, and extracted using Qiagen's DNeasy Blood & Tissue kit. Sample libraries were prepared using the Illumina DNA Prep kit and IDT 10-bp UDI indices, and sequenced on an Illumina NextSeq 2000, producing 2x151bp reads. Demultiplexing, quality control and adapter trimming was performed with BCL Convert (Illumina, Inc.). Illumina reads were also mapped to the classical *S.* Typhi reference strain CT18 (GenBank Accession AL513382.1) using Snippy v4.6.0 [1] for subsequent genotype assignment using GenoTyphi v1.9.1 [2,3]. To generate draft Illumina only assemblies, Illumina reads were quality filtered and trimmed using Trimmomatic v0.38 [4] and assembled *de novo* using SPAdes v3.14.1 [5] in Python v2.7.14. The final draft assemblies were filtered to contain only contigs ≥500 bp in length and with ≥5x k-mer coverage, as previously described [6]. To generate closed complete genomes of the isolates from suspected carrier clusters, Nanopore samples were prepared for sequencing using Oxford Nanopore Technologies (ONT) “Genomic DNA by Ligation” kit and protocol (Oxford Nanopore Technologies—ONT, Oxford, UK). All samples were run on Nanopore R9 flow cells and Guppy v4.4.2 [7] was used for base calling. Quality control and adapter trimming was performed with bcl2fastq v2.20.0.445 (Illumina, Inc.) and porechop v0.2.3_seqan2.1.1 [8] for Illumina and ONT sequencing, respectively. Hybrid assembly with Illumina and ONT reads was performed with Unicycler v0.4.8 [9] and resulting assembly statistics were recorded with QUAST v5.0.2 [10]. Default parameters were used for all software tools unless otherwise specified. All raw reads and assembled data were submitted to NCBI and Accession numbers are provided in Table S1.

**Phylogenetic analysis**

Core genome single nucleotide polymorphisms (SNPs) were called against a local complete S. Typhi reference isolate 1521-2017_CI (GenBank Accession CP120397) using NASP v1.2.0 [11]. To exclude regions of recombination, the resultant alignment was analyzed with Gubbins v2.4.1 [12]. RAxML v8.2.10 [13] was run on the PHYLIP format alignment of filtered polymorphic sites using the generalized time-reversible (GTR) nucleotide substitution model with a Gamma distribution and the Lewis ascertainment bias correction (ASC_GTRGAMMA in RAxML) and 100 bootstrap pseudo-replicates. The resulting ML phylogeny tree was visualized in iTOL [14], mid-point rooted, and decorated with genotype and epidemiologic variables relating origin, travel-associations, and carrier clusters.

**SNP calculations**

Pairwise SNP distances between core genomes without recombination sites were calculated using snp-dists v0.8.2 [15] and the resultant matrix is in Table S2. The molten (list) output option was also selected in order to compare SNP differences among epidemiologically linked isolates.

**Plasmid Replicons and Antimicrobial Susceptibility Testing**

Pathogenwatch is an online bacterial genome surveillance tool for *S.* Typhi [16,17] with *in silico* typing modules for uploaded data and a global collection of published *S.* Typhi assemblies for comparison [17]. All genome assemblies from this were uploaded to Pathogenwatch for plasmid replicons detection using the PlasmidFinder [18] Enterobacteriaceae database implemented as “IncTyper” in Pathogenwatch. Genomic predictions of AMR were determined using the Pathogenwatch curated AMR library, which includes both genes and point mutations known to confer phenotypic resistances [16]. Phenotypic antimicrobial susceptibility testing was performed using the Kirby-Bauer disc diffusion method [19] and CLSI breakpoints at the Instituto de Salud Pública de Chile (Institute of Public Health, Chile).

REFERENCES

1. Seemann T. Snippy (v4.6.0), GitHub https://github.com/tseemann/snippy. 2020.

2. Wong VK, Baker S, Connor TR, et al. An extended genotyping framework for *Salmonella enterica* serovar Typhi, the cause of human typhoid. Nat Commun. Nature Publishing Group; **2016**; 7(1):12827.

3. Dyson ZA, Holt KE. Five years of GenoTyphi: Updates to the global *Salmonella* Typhi genotyping framework. J Infect Dis. **2021**; 224(Suppl 7):S775–S780.

4. Bolger AM, Lohse M, Usadel B. Trimmomatic: a flexible trimmer for Illumina sequence data. Bioinformatics. **2014**; 30(15):2114–2120.

5. Bankevich A, Nurk S, Antipov D, et al. SPAdes: A new genome assembly algorithm and its applications to single-cell sequencing. J Comput Biol. **2012**; 19(5):455–477.

6. Hazen TH, Nagaraj S, Sen S, et al. Genome and functional characterization of colonization factor antigen I- and CS6-encoding heat-stable enterotoxin-only enterotoxigenic *Escherichia coli* reveals lineage and geographic variation. mSystems. **2019**; 4(1):e00329-18.

7. Oxford Nanopore Technologies. Guppy v4.4.2 [Internet]. 2021. Available from: https://pypi.org/project/ont-pyguppy-client-lib/

8. Wick RR. Porechop v0.2.4 (2018), GitHub repository, https://github.com/rrwick/Porechop.

9. Wick RR, Judd LM, Gorrie CL, Holt KE. Unicycler: Resolving bacterial genome assemblies from short and long sequencing reads. PLoS Comput Biol. **2017**; 13(6):1–22.

10. Gurevich A, Saveliev V, Vyahhi N, Tesler G. QUAST: Quality assessment tool for genome assemblies. Bioinformatics. **2013**; 29(8):1072–1075.

11. Sahl JW, Lemmer D, Travis J, et al. NASP: An accurate, rapid method for the identification of SNPs in WGS datasets that supports flexible input and output formats. Microb Genomics. **2016**; 2(8):e000074.

12. Croucher NJ, Page AJ, Connor TR, et al. Rapid phylogenetic analysis of large samples of recombinant bacterial whole genome sequences using Gubbins. Nucleic Acids Res. **2015**; 43(3):e15.

13. Stamatakis A. RAxML version 8: A tool for phylogenetic analysis and post-analysis of large phylogenies. Bioinformatics. **2014**; 30(9):1312–1313.

14. Letunic I, Bork P. Interactive Tree Of Life (iTOL) v4: Recent updates and new developments. Nucleic Acids Res. **2019**; 47:W256–W259.

15. Seeman T. Snp-dists v0.8.2 [Internet]. GitHub; 2021. Available from: https://github.com/tseemann/snp-dists

16. Argimón S, Yeats CA, Goater RJ, et al. A global resource for genomic predictions of antimicrobial resistance and surveillance of *Salmonella* Typhi at Pathogenwatch. Nat Commun. Springer US; **2021**; 12(1):2879.

17. Centre for Genomic Pathogen Surveillance (CGPS). Pathogenwatch Technical Descriptions. https://cgps.gitbook.io/pathogenwatch/technical-descriptions. Accessed 10 December 2021.

18. Carattoli A, Zankari E, García-Fernández A, et al. *In silico* detection and typing of plasmids using PlasmidFinder and plasmid multilocus sequence typing. Antimicrob Agents Chemother. **2014**; 58(7):3895–3903.

19. Bauer AW, Kirby WMM, Sherris JC, Turck M. Antibiotic susceptibility testing by a standardized single disk method. Am J Clin Pathol. **1966**; 45(4):493–496.
